# 1-Acetyl-2-*r*,6-*c*-bis­(4-chloro­phen­yl)-3-methyl-1,2,5,6-tetra­hydro­pyridin-4-yl acetate

**DOI:** 10.1107/S1600536810047586

**Published:** 2010-11-20

**Authors:** V. Vimalraj, K. Pandiarajan

**Affiliations:** a Department of Chemistry, Annamalai University, Annamalai Nagar 608 002, Tamilnadu, India

## Abstract

In the title compound, C_22_H_21_Cl_2_NO_3_, the pyridine ring adopts a half-chair conformation and the 4-chloro­phenyl groups occupy axial positions. The 4-chloro­phenyl groups are almost perpendicular to the plane of the tetra­hydro­pyridine ring forming dihedral angles 84.62 (6) and 85.55 (5)°; the dihedral angle between the two 4-chloro­phenyl rings is 12.16 (4)°. The crystal structure is stabilized by inter­molecular C—H⋯O inter­actions.

## Related literature

For a related structure, see: Subha Nandhini *et al.* (2003 ▶).
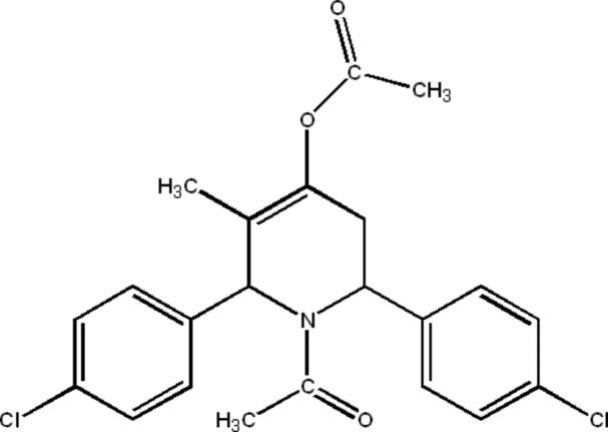


## Experimental

### 

#### Crystal data

C_22_H_21_Cl_2_NO_3_
*M* *_r_* = 418.30
Monoclinic, 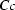

*a* = 16.560 (3) Å
*b* = 14.809 (3) Å
*c* = 10.241 (2) Å
β = 124.27 (3)°
*V* = 2075.5 (10) Å^3^
*Z* = 4
Mo *K*α radiation
μ = 0.34 mm^−1^
*T* = 293 K
0.30 × 0.25 × 0.20 mm

#### Data collection

Bruker Kappa APEXII CCD diffractometer
Absorption correction: multi-scan (*SADABS*; Bruker, 1999 ▶) *T*_min_ = 0.866, *T*_max_ = 0.936
13520 measured reflections
5546 independent reflections
4578 reflections with *I* > 2σ(*I*)
*R*_int_ = 0.020

#### Refinement

*R*[*F* ^2^ > 2σ(*F* ^2^)] = 0.037
*wR*(*F* ^2^) = 0.100
*S* = 1.04
5546 reflections
256 parameters
2 restraints
H-atom parameters constrained
Δρ_max_ = 0.27 e Å^−3^
Δρ_min_ = −0.17 e Å^−3^
Absolute structure: Flack (1983 ▶), 2649 Friedel pairs
Flack parameter: 0.02 (5)

### 

Data collection: *APEX2* (Bruker, 2004 ▶); cell refinement: *APEX2* and *SAINT* (Bruker, 2004 ▶); data reduction: *SAINT*; program(s) used to solve structure: *SIR92* (Altomare *et al.*, 1993 ▶); program(s) used to refine structure: *SHELXL97* (Sheldrick, 2008 ▶); molecular graphics: *ORTEP-3* (Farrugia, 1997 ▶) and *Mercury* (Bruno *et al.*, 2002 ▶); software used to prepare material for publication: *PLATON* (Spek, 2009 ▶).

## Supplementary Material

Crystal structure: contains datablocks I, global. DOI: 10.1107/S1600536810047586/pv2343sup1.cif

Structure factors: contains datablocks I. DOI: 10.1107/S1600536810047586/pv2343Isup2.hkl

Additional supplementary materials:  crystallographic information; 3D view; checkCIF report

## Figures and Tables

**Table 1:** Hydrogen-bond geometry (Å, °)

*D*—H⋯*A*	*D*—H	H⋯*A*	*D*⋯*A*	*D*—H⋯*A*
C3—H3⋯O3^i^	0.98	2.44	3.341 (3)	152
C4—H4*B*⋯O1^ii^	0.97	2.35	3.308 (3)	169

Symmetry codes: (i) 
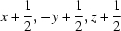
; (ii) 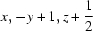.

**Fig. 1.**
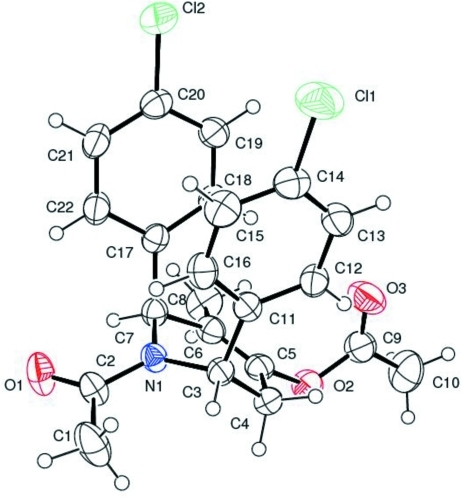
The molecular structure of the title compound, showing 50% probability displacement ellipsoids.

**Fig. 2.**
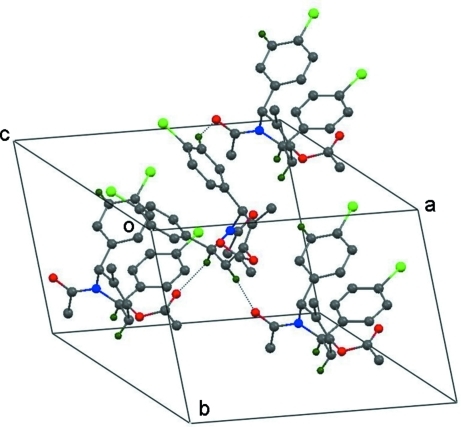
Part of the crystal structure showing the formation of the possible three C—H···O hydrogen bonds C4—H4B···O1i, C3—H3···O3ii and C21—H21···O1iii [symmetry code: (i) x, -y+1, z+1/2, (ii) x+1/2, -y+1/2, z+1/2 /and (iii) x,-y+1,+z-1/2] with in the unit cell.

## supplementary crystallographic information

### Comment

The X-ray crystal structure determination of the title compound was undertaken to determine the effect of substitution of acetyl and acetoxy groups at positions 1 and 4, respectively, on the conformation of the tetrahydropyridine ring. The tetrahydropyridine ring adopts a half chair conformation with N1 and C3 atoms 0.324 (3) and -0.328 (3) Å, respectively, out of the basal plane formed by the remaining ring atoms (C4/C5/C6/C7) and the aryl groups occupy axial positions. The 4-chlorophenyl groups, C11–C16/Cl1 and C17–C22/Cl2, are almost perpendicular to the tetrahydropyridine ring forming dihedral angles 84.62 (6) and 85.55 (5)°, respectively; the dihedral angle between the two 4-chlorophenyl rings is 12.16 (4)°. The aryl groups take axial positions to avoid A1,3 strain. The acetoxy group O2/O3/C9/C10 is almost perpendicular (88.05 (6)°) to the tetrahydropyridine ring. The crystal structure is stabilized by intermolecular C—H···O interactions. The bond distances and angles in the title compound are comparable to a similar structure reported earlier (Subha Nandhini *et al.*, (2003).

### Experimental

A mixture of 3 t-methyl-2r,6c-bis(4-chlorophenyl)-piperidin-4-one (0.01 mol) and hippuric acid in acetic anhydride (20 ml) was refluxed for about 2 h. After the completion of reaction, excess of acetic anhydride was removed by distillation and water (50 ml) was added. The title compound thus obtained as a solid product was separated and colourless crystals were grown by slow evaporation method using ethanol as solvent.

### Refinement

The H atoms were included in the refinement at geometrically idealized positions with C—H distances 0.93, 0.96, 0.97 and 0.99 Å for aryl, methyl, methylene and methyne type H-atoms in riding mode allowing *U*_iso_(H) = 1.5 or 1.2 *U*_eq_ of the carrier methyl and non-methyl C-atoms, respectively.

### Crystal data

**Table:**

C_22_H_21_Cl_2_NO_3_	*F*(000) = 872
*M**_r_* = 418.30	*D*_x_ = 1.339 Mg m^−^^3^
Monoclinic, *C**c*	Melting point: 411 K
Hall symbol: C -2yc	Mo *K*α radiation, λ = 0.71073 Å
*a* = 16.560 (3) Å	Cell parameters from 6663 reflections
*b* = 14.809 (3) Å	θ = 2.8–59.2°
*c* = 10.241 (2) Å	µ = 0.34 mm^−^^1^
β = 124.27 (3)°	*T* = 293 K
*V* = 2075.5 (10) Å^3^	Block, colourless
*Z* = 4	0.30 × 0.25 × 0.20 mm

### Data collection

**Table:**

Bruker Kappa APEXII CCD diffractometer	5546 independent reflections
Radiation source: fine-focus sealed tube	4578 reflections with *I* > 2σ(*I*)
graphite	*R*_int_ = 0.020
ω and φ scans	θ_max_ = 29.6°, θ_min_ = 2.0°
Absorption correction: multi-scan (*SADABS*; Bruker, 1999)	*h* = −22→22
*T*_min_ = 0.866, *T*_max_ = 0.936	*k* = −20→20
13520 measured reflections	*l* = −14→14

### Refinement

**Table:**

Refinement on *F*^2^	Secondary atom site location: difference Fourier map
Least-squares matrix: full	Hydrogen site location: inferred from neighbouring sites
*R*[*F*^2^ > 2σ(*F*^2^)] = 0.037	H-atom parameters constrained
*wR*(*F*^2^) = 0.100	*w* = 1/[σ^2^(*F*_o_^2^) + (0.0528*P*)^2^ + 0.2729*P*] where *P* = (*F*_o_^2^ + 2*F*_c_^2^)/3
*S* = 1.04	(Δ/σ)_max_ = 0.011
5546 reflections	Δρ_max_ = 0.27 e Å^−^^3^
256 parameters	Δρ_min_ = −0.17 e Å^−^^3^
2 restraints	Absolute structure: Flack (1983), 2649 Friedel pairs
Primary atom site location: structure-invariant direct methods	Flack parameter: 0.02 (5)

### Special details

**Table:**

Geometry. All e.s.d.'s (except the e.s.d. in the dihedral angle between two l.s. planes) are estimated using the full covariance matrix. The cell e.s.d.'s are taken into account individually in the estimation of e.s.d.'s in distances, angles and torsion angles; correlations between e.s.d.'s in cell parameters are only used when they are defined by crystal symmetry. An approximate (isotropic) treatment of cell e.s.d.'s is used for estimating e.s.d.'s involving l.s. planes.
Refinement. Refinement of *F*^2^ against ALL reflections. The weighted *R*-factor *wR* and goodness of fit *S* are based on *F*^2^, conventional *R*-factors *R* are based on *F*, with *F* set to zero for negative *F*^2^. The threshold expression of *F*^2^ > σ(*F*^2^) is used only for calculating *R*-factors(gt) *etc*. and is not relevant to the choice of reflections for refinement. *R*-factors based on *F*^2^ are statistically about twice as large as those based on *F*, and *R*- factors based on ALL data will be even larger.

### Fractional atomic coordinates and isotropic or equivalent isotropic displacement parameters (Å^2^)

**Table:**

	*x*	*y*	*z*	*U*_iso_*/*U*_eq_	
Cl2	0.12844 (5)	0.18128 (4)	−0.47636 (7)	0.07210 (17)	
Cl1	0.25477 (6)	−0.00598 (5)	−0.08332 (9)	0.0858 (2)	
O2	0.03552 (10)	0.37134 (10)	0.21164 (17)	0.0576 (4)	
N1	0.26481 (10)	0.40891 (9)	0.16833 (17)	0.0389 (3)	
C2	0.33771 (12)	0.46887 (12)	0.2128 (2)	0.0478 (4)	
O1	0.32636 (10)	0.53254 (10)	0.1281 (2)	0.0664 (4)	
C20	0.13277 (14)	0.25613 (13)	−0.3423 (2)	0.0488 (4)	
C17	0.14951 (12)	0.36840 (12)	−0.1132 (2)	0.0395 (3)	
O3	−0.01433 (13)	0.24011 (13)	0.0838 (2)	0.0784 (5)	
C19	0.08085 (13)	0.23746 (14)	−0.2795 (2)	0.0496 (4)	
H19	0.0403	0.1871	−0.3131	0.059*	
C3	0.27461 (12)	0.33000 (11)	0.2627 (2)	0.0398 (3)	
H3	0.3404	0.3321	0.3604	0.048*	
C7	0.16844 (11)	0.42629 (12)	0.0235 (2)	0.0398 (3)	
H7	0.1680	0.4894	−0.0058	0.048*	
C4	0.20259 (13)	0.33908 (12)	0.3092 (2)	0.0457 (4)	
H4A	0.1924	0.2803	0.3393	0.055*	
H4B	0.2301	0.3785	0.4003	0.055*	
C11	0.26760 (11)	0.24311 (10)	0.17759 (19)	0.0391 (3)	
C18	0.08892 (12)	0.29388 (13)	−0.1653 (2)	0.0442 (4)	
H18	0.0531	0.2815	−0.1229	0.053*	
C21	0.19096 (16)	0.33134 (13)	−0.2975 (3)	0.0539 (5)	
H21	0.2246	0.3444	−0.3435	0.065*	
C16	0.32496 (13)	0.23387 (13)	0.1197 (2)	0.0492 (4)	
H16	0.3668	0.2807	0.1349	0.059*	
C15	0.32233 (15)	0.15862 (14)	0.0411 (3)	0.0557 (5)	
H15	0.3611	0.1545	0.0022	0.067*	
C5	0.10804 (13)	0.37564 (13)	0.1803 (2)	0.0446 (4)	
C12	0.20837 (14)	0.17175 (12)	0.1571 (2)	0.0462 (4)	
H12	0.1699	0.1757	0.1966	0.055*	
C22	0.19884 (14)	0.38696 (13)	−0.1840 (2)	0.0497 (4)	
H22	0.2380	0.4382	−0.1537	0.060*	
C6	0.08920 (12)	0.41668 (12)	0.0530 (2)	0.0437 (4)	
C14	0.26133 (15)	0.08872 (13)	0.0203 (2)	0.0518 (4)	
C13	0.20498 (15)	0.09451 (14)	0.0792 (2)	0.0536 (4)	
H13	0.1648	0.0467	0.0666	0.064*	
C9	−0.02127 (14)	0.29704 (16)	0.1591 (3)	0.0569 (5)	
C8	−0.00692 (15)	0.45758 (17)	−0.0687 (3)	0.0646 (5)	
H8A	−0.0584	0.4164	−0.0930	0.097*	
H8B	−0.0091	0.4697	−0.1627	0.097*	
H8C	−0.0152	0.5130	−0.0289	0.097*	
C1	0.43369 (16)	0.45659 (18)	0.3692 (3)	0.0773 (7)	
H1A	0.4787	0.5024	0.3819	0.116*	
H1B	0.4598	0.3981	0.3727	0.116*	
H1C	0.4239	0.4615	0.4528	0.116*	
C10	−0.0915 (2)	0.2967 (2)	0.2046 (4)	0.0833 (8)	
H10A	−0.1459	0.3350	0.1341	0.125*	
H10B	−0.0599	0.3187	0.3108	0.125*	
H10C	−0.1143	0.2363	0.1985	0.125*	

### Atomic displacement parameters (Å^2^)

**Table:**

	*U*^11^	*U*^22^	*U*^33^	*U*^12^	*U*^13^	*U*^23^
Cl2	0.0966 (4)	0.0705 (3)	0.0709 (3)	−0.0064 (3)	0.0603 (3)	−0.0127 (3)
Cl1	0.1101 (5)	0.0630 (3)	0.0896 (4)	0.0059 (3)	0.0595 (4)	−0.0197 (3)
O2	0.0616 (8)	0.0641 (9)	0.0680 (9)	−0.0104 (7)	0.0490 (8)	−0.0149 (7)
N1	0.0354 (6)	0.0352 (6)	0.0391 (7)	−0.0014 (6)	0.0166 (6)	0.0038 (6)
C2	0.0403 (9)	0.0400 (9)	0.0554 (11)	−0.0051 (7)	0.0222 (9)	−0.0003 (8)
O1	0.0557 (8)	0.0515 (8)	0.0809 (11)	−0.0070 (6)	0.0317 (8)	0.0186 (8)
C20	0.0537 (10)	0.0509 (10)	0.0419 (9)	0.0050 (9)	0.0269 (9)	0.0031 (8)
C17	0.0355 (7)	0.0441 (9)	0.0363 (8)	0.0034 (7)	0.0186 (7)	0.0069 (7)
O3	0.0740 (10)	0.0830 (11)	0.0867 (12)	−0.0323 (9)	0.0504 (10)	−0.0365 (10)
C19	0.0419 (9)	0.0561 (11)	0.0468 (10)	−0.0072 (8)	0.0226 (8)	−0.0041 (8)
C3	0.0401 (8)	0.0393 (8)	0.0349 (8)	−0.0014 (7)	0.0179 (7)	0.0031 (6)
C7	0.0383 (8)	0.0390 (8)	0.0402 (9)	0.0005 (7)	0.0211 (8)	0.0046 (7)
C4	0.0556 (10)	0.0439 (9)	0.0417 (9)	−0.0039 (8)	0.0299 (9)	−0.0022 (7)
C11	0.0364 (8)	0.0363 (8)	0.0362 (8)	0.0031 (6)	0.0154 (7)	0.0054 (6)
C18	0.0396 (8)	0.0535 (10)	0.0425 (9)	−0.0022 (7)	0.0249 (8)	0.0005 (8)
C21	0.0649 (11)	0.0550 (11)	0.0584 (11)	0.0003 (9)	0.0447 (10)	0.0088 (9)
C16	0.0416 (9)	0.0492 (9)	0.0576 (11)	0.0014 (8)	0.0284 (9)	0.0054 (8)
C15	0.0552 (11)	0.0560 (11)	0.0627 (12)	0.0108 (9)	0.0372 (10)	0.0038 (9)
C5	0.0480 (9)	0.0459 (9)	0.0488 (10)	−0.0057 (7)	0.0326 (8)	−0.0114 (8)
C12	0.0522 (9)	0.0416 (9)	0.0519 (10)	0.0010 (8)	0.0335 (9)	0.0043 (8)
C22	0.0562 (10)	0.0459 (10)	0.0548 (11)	−0.0036 (8)	0.0360 (10)	0.0056 (8)
C6	0.0387 (8)	0.0449 (9)	0.0479 (10)	−0.0021 (7)	0.0246 (8)	−0.0063 (8)
C14	0.0596 (11)	0.0439 (9)	0.0473 (10)	0.0126 (8)	0.0273 (9)	0.0026 (8)
C13	0.0600 (11)	0.0434 (10)	0.0539 (11)	−0.0042 (8)	0.0299 (10)	−0.0004 (8)
C9	0.0465 (10)	0.0690 (13)	0.0547 (11)	−0.0071 (9)	0.0283 (10)	−0.0039 (10)
C8	0.0464 (10)	0.0793 (15)	0.0663 (13)	0.0130 (10)	0.0305 (10)	0.0061 (11)
C1	0.0444 (11)	0.0672 (15)	0.0794 (17)	−0.0147 (10)	0.0100 (11)	0.0094 (12)
C10	0.0653 (14)	0.105 (2)	0.100 (2)	−0.0069 (15)	0.0590 (16)	0.0042 (17)

### Geometric parameters (Å, °)

**Table:**

Cl2—C20	1.7343 (19)	C11—C16	1.381 (2)
Cl1—C14	1.725 (2)	C18—H18	0.9300
O2—C9	1.347 (3)	C21—C22	1.369 (3)
O2—C5	1.409 (2)	C21—H21	0.9300
N1—C2	1.354 (2)	C16—C15	1.361 (3)
N1—C7	1.465 (2)	C16—H16	0.9300
N1—C3	1.466 (2)	C15—C14	1.377 (3)
C2—O1	1.222 (2)	C15—H15	0.9300
C2—C1	1.503 (3)	C5—C6	1.307 (3)
C20—C19	1.361 (2)	C12—C13	1.378 (3)
C20—C21	1.372 (3)	C12—H12	0.9300
C17—C18	1.381 (3)	C22—H22	0.9300
C17—C22	1.391 (2)	C6—C8	1.490 (3)
C17—C7	1.516 (2)	C14—C13	1.369 (3)
O3—C9	1.191 (3)	C13—H13	0.9300
C19—C18	1.381 (3)	C9—C10	1.475 (3)
C19—H19	0.9300	C8—H8A	0.9600
C3—C4	1.519 (2)	C8—H8B	0.9600
C3—C11	1.521 (2)	C8—H8C	0.9600
C3—H3	0.9800	C1—H1A	0.9600
C7—C6	1.510 (2)	C1—H1B	0.9600
C7—H7	0.9800	C1—H1C	0.9600
C4—C5	1.470 (3)	C10—H10A	0.9600
C4—H4A	0.9700	C10—H10B	0.9600
C4—H4B	0.9700	C10—H10C	0.9600
C11—C12	1.375 (2)		
			
C9—O2—C5	116.06 (15)	C15—C16—H16	118.9
C2—N1—C7	118.79 (14)	C11—C16—H16	118.9
C2—N1—C3	123.66 (14)	C16—C15—C14	118.94 (18)
C7—N1—C3	117.42 (13)	C16—C15—H15	120.5
O1—C2—N1	121.05 (17)	C14—C15—H15	120.5
O1—C2—C1	119.84 (18)	C6—C5—O2	119.56 (17)
N1—C2—C1	119.11 (17)	C6—C5—C4	127.06 (15)
C19—C20—C21	121.22 (17)	O2—C5—C4	113.09 (16)
C19—C20—Cl2	119.27 (15)	C11—C12—C13	121.28 (17)
C21—C20—Cl2	119.48 (14)	C11—C12—H12	119.4
C18—C17—C22	117.96 (17)	C13—C12—H12	119.4
C18—C17—C7	122.41 (14)	C21—C22—C17	121.33 (18)
C22—C17—C7	119.52 (16)	C21—C22—H22	119.3
C20—C19—C18	119.45 (17)	C17—C22—H22	119.3
C20—C19—H19	120.3	C5—C6—C8	124.46 (16)
C18—C19—H19	120.3	C5—C6—C7	119.81 (15)
N1—C3—C4	108.74 (14)	C8—C6—C7	115.73 (16)
N1—C3—C11	110.61 (13)	C13—C14—C15	120.48 (18)
C4—C3—C11	115.75 (14)	C13—C14—Cl1	119.96 (17)
N1—C3—H3	107.1	C15—C14—Cl1	119.55 (15)
C4—C3—H3	107.1	C14—C13—C12	119.44 (18)
C11—C3—H3	107.1	C14—C13—H13	120.3
N1—C7—C6	110.78 (13)	C12—C13—H13	120.3
N1—C7—C17	112.11 (14)	O3—C9—O2	122.43 (18)
C6—C7—C17	112.35 (14)	O3—C9—C10	125.6 (2)
N1—C7—H7	107.1	O2—C9—C10	111.9 (2)
C6—C7—H7	107.1	C6—C8—H8A	109.5
C17—C7—H7	107.1	C6—C8—H8B	109.5
C5—C4—C3	112.22 (14)	H8A—C8—H8B	109.5
C5—C4—H4A	109.2	C6—C8—H8C	109.5
C3—C4—H4A	109.2	H8A—C8—H8C	109.5
C5—C4—H4B	109.2	H8B—C8—H8C	109.5
C3—C4—H4B	109.2	C2—C1—H1A	109.5
H4A—C4—H4B	107.9	C2—C1—H1B	109.5
C12—C11—C16	117.53 (16)	H1A—C1—H1B	109.5
C12—C11—C3	123.71 (15)	C2—C1—H1C	109.5
C16—C11—C3	118.74 (15)	H1A—C1—H1C	109.5
C17—C18—C19	120.89 (15)	H1B—C1—H1C	109.5
C17—C18—H18	119.6	C9—C10—H10A	109.5
C19—C18—H18	119.6	C9—C10—H10B	109.5
C22—C21—C20	119.07 (16)	H10A—C10—H10B	109.5
C22—C21—H21	120.5	C9—C10—H10C	109.5
C20—C21—H21	120.5	H10A—C10—H10C	109.5
C15—C16—C11	122.30 (17)	H10B—C10—H10C	109.5
			
C7—N1—C2—O1	−5.9 (3)	Cl2—C20—C21—C22	−175.81 (16)
C3—N1—C2—O1	178.46 (18)	C12—C11—C16—C15	1.7 (3)
C7—N1—C2—C1	173.89 (19)	C3—C11—C16—C15	−179.45 (18)
C3—N1—C2—C1	−1.8 (3)	C11—C16—C15—C14	−0.8 (3)
C21—C20—C19—C18	−1.8 (3)	C9—O2—C5—C6	93.2 (2)
Cl2—C20—C19—C18	175.93 (15)	C9—O2—C5—C4	−92.5 (2)
C2—N1—C3—C4	117.77 (18)	C3—C4—C5—C6	−15.7 (3)
C7—N1—C3—C4	−57.97 (18)	C3—C4—C5—O2	170.56 (14)
C2—N1—C3—C11	−114.08 (18)	C16—C11—C12—C13	−1.2 (3)
C7—N1—C3—C11	70.18 (18)	C3—C11—C12—C13	−179.91 (18)
C2—N1—C7—C6	−130.70 (16)	C20—C21—C22—C17	0.3 (3)
C3—N1—C7—C6	45.2 (2)	C18—C17—C22—C21	−2.6 (3)
C2—N1—C7—C17	102.91 (18)	C7—C17—C22—C21	173.71 (17)
C3—N1—C7—C17	−81.14 (17)	O2—C5—C6—C8	−2.5 (3)
C18—C17—C7—N1	103.99 (18)	C4—C5—C6—C8	−175.8 (2)
C22—C17—C7—N1	−72.1 (2)	O2—C5—C6—C7	176.44 (15)
C18—C17—C7—C6	−21.5 (2)	C4—C5—C6—C7	3.1 (3)
C22—C17—C7—C6	162.36 (16)	N1—C7—C6—C5	−16.0 (2)
N1—C3—C4—C5	39.76 (19)	C17—C7—C6—C5	110.29 (17)
C11—C3—C4—C5	−85.43 (19)	N1—C7—C6—C8	163.06 (16)
N1—C3—C11—C12	−131.11 (17)	C17—C7—C6—C8	−70.7 (2)
C4—C3—C11—C12	−6.9 (2)	C16—C15—C14—C13	−0.7 (3)
N1—C3—C11—C16	50.2 (2)	C16—C15—C14—Cl1	178.36 (16)
C4—C3—C11—C16	174.38 (15)	C15—C14—C13—C12	1.2 (3)
C22—C17—C18—C19	2.7 (3)	Cl1—C14—C13—C12	−177.82 (16)
C7—C17—C18—C19	−173.48 (16)	C11—C12—C13—C14	−0.3 (3)
C20—C19—C18—C17	−0.6 (3)	C5—O2—C9—O3	−3.6 (3)
C19—C20—C21—C22	1.9 (3)	C5—O2—C9—C10	177.1 (2)

### Hydrogen-bond geometry (Å, °)

**Table:**

*D*—H···*A*	*D*—H	H···*A*	*D*···*A*	*D*—H···*A*
C3—H3···O3^i^	0.98	2.44	3.341 (3)	152
C4—H4B···O1^ii^	0.97	2.35	3.308 (3)	169
C7—H7···O1	0.98	2.26	2.701 (2)	106

Symmetry codes: (i) *x*+1/2, −*y*+1/2, *z*+1/2; (ii) *x*, −*y*+1, *z*+1/2.
